# A protocol for the impact of Tongmai Jiangtang capsules on the risk of cardiovascular and cerebrovascular events in metabolic syndrome

**DOI:** 10.3389/fcvm.2026.1817298

**Published:** 2026-07-28

**Authors:** Anxiang Li, Yuntao Ma, Zhuhong Chen, Qiyun Lu, Chaolan Liu, Peng Zhou, Qingshun Liang, Yiming Tao, Meilin Chen, Zihan Chen, Fan Luo, Xingxing Lei, Shulin Liu, Jieer Long, Hequn Xiao, Chongsong Cui, Qiqi Ren, Yunwei Liu, Tuoling Zhong, Jiamiao Liu, Guanjie Fan, Ya Liu, Zhenliang Hui, Qiu Chen, Zhenjie Liu

**Affiliations:** 1 Department of Endocrinology, Guangdong Provincial Hospital of Chinese Medicine, Guangzhou, China; 2 The Second Affiliated Hospital of Guangzhou University of Chinese Medicine, Guangzhou, China; 3 Guangzhou University of Traditional Chinese Medicine, Guangzhou, China; 4 First Teaching Hospital of Tianjin University of Traditional Chinese Medicine, Tianjin, China; 5 Xiyuan Hospital of China Academy of Chinese Medical Sciences, Beijing, China; 6 The First Affiliated Hospital of Guangzhou University of Chinese Medicine, Guangzhou, China; 7 Yinchuan Brain-Heart Integrated Internet Hospital Co., Ltd., Yinchuan, China; 8 Shanxi Provincial Hospital of Chinese Medicine, Xi’an, China; 9 Traditional Chinese Medicine Hospital of Sichuan Province, Chengdu, Sichuan, China

**Keywords:** cardiovascular and cerebrovascular events, Chinese medicine, metabolic syndrome, randomized controlled trial, Tongmai Jiangtang capsules

## Abstract

**Background:**

Metabolic syndrome (MetS) significantly increases the risk of major adverse cardiovascular events (MACE), yet evidence-based complementary therapies remain limited. Tongmai Jiangtang Capsule (TJC), a traditional Chinese medicine (TCM) compound, has demonstrated benefits for glycolipid metabolism and microvascular complications, but its effect on hard cardiovascular and cerebrovascular (CCV) endpoints remains unconfirmed.

**Objective:**

To explore whether TJC, as an adjunctive therapy to standard care, reduces the incidence of MACE in patients with MetS and Qi deficiency-blood stasis syndrome.

**Methods:**

This is a randomized, double-blind, placebo-controlled, multicenter trial. A total of 530 participants aged ≥65 years, diagnosed with MetS and the TCM pattern of Qi Deficiency and Blood Stasis, and at high CCV risk, will be enrolled. They will be randomly assigned (1:1) to receive either TJC or a matched placebo, in addition to standard care, for 26 weeks, followed by a 26-week follow-up. The primary endpoint is the composite of 3P-MACE (cardiovascular death, non-fatal myocardial infarction, non-fatal stroke). Secondary endpoints include metabolic parameters, vascular assessments, TCM symptom scores, and quality of life. Omics analyses will explore potential mechanisms. The trial was registered on ClinicalTrials.gov (NCT06908473).

**Discussion:**

This rigorously designed trial is expected to provide high-level evidence regarding the role of TJC in preventing CCV events in MetS. If positive, it will offer a novel, multi-target integrative treatment strategy. The findings will also advance the evidence-based evaluation of TCM compounds and contribute to chronic disease prevention goals. Regardless of the primary outcome, the mechanistic insights will inform future research on formula optimization and precision intervention.

**Clinical Trial Registration:**

https://clinicaltrials.gov/expert-search?term=NCT06908473, identifier, NCT06908473.

## Introduction

Metabolic Syndrome (MetS) represents a significant global public health challenge in the 21st century (1). It is characterized by a cluster of core components including central obesity, hyperglycemia, hypertension, and dyslipidemia (elevated triglycerides/reduced high-density lipoprotein cholesterol) (2). Its pathophysiological essence involves the superimposition of insulin resistance (IR)-mediated metabolic disorders and a state of chronic low-grade inflammation (3–5). Since its first formal definition by the World Health Organization (WHO) in 1998 (6), the prevalence of MetS has experienced exponential growth, paralleling the global rise in obesity rates and population aging. The latest Chinese epidemiological data (2023 Report on Chronic Diseases and Risk Factors Surveillance in China) indicates that the prevalence of MetS among adults has reached 31.2%, with a notable trend towards younger age groups—the prevalence among individuals under 40 has increased by 47% compared to a decade ago. This population faces not only a high risk of metabolic diseases such as diabetes and non-alcoholic fatty liver disease but also (7–9), due to vascular endothelial injury and accelerated atherosclerosis induced by metabolic disturbances, constitutes a “high-risk group” for cardiovascular and cerebrovascular events (5, 10–12) (e.g., myocardial infarction, stroke). Studies have shown that the risk of cardiovascular events in patients with MetS is 2.3-3.1 times that of healthy individuals, with all-cause mortality increased by 1.8-2.5 times; among these, MetS patients with comorbid diabetes have a 10-year risk of cardiovascular and cerebrovascular events exceeding 40% (13–15). However, current clinical management of MetS predominantly focuses on intervening in individual risk factors (e.g., glucose-lowering, blood pressure control, lipid regulation), lacking multi-target intervention strategies aimed at the “metabolic-vascular injury” network (16). Furthermore, some pharmacological agents (e.g., long-term use of statins, insulin) are associated with side effects such as hepatic and renal impairment and hypoglycemia, making it difficult to meet patients’ needs for “safe, efficient, and holistic regulation” therapeutic approaches. Recent large cardiovascular outcome trials (17, 18) have shown that in high-risk populations with obesity and cardiovascular disease, the annual MACE rate remains substantial despite contemporary standard care.

Traditional Chinese medicine (TCM) compounds, by virtue of their “multi-component, multi-target, multi-pathway” intervention characteristics, offer novel insights for the comprehensive management of MetS. Meta-analyses have shown that TCM or the combination of TCM and Western medicine can effectively improve metabolic syndrome and reduce cardiovascular and cerebrovascular risk (19–21). Tongmai Jiangtang Capsule (TJC) is an innovative proprietary Chinese medicine developed independently, derived from the classical empirical formula “Method for Boosting Qi, Activating Blood, and Dredging Collaterals.” Its main components include Astragalus membranaceus (Huangqi), Salvia miltiorrhiza (Danshen), Ligusticum chuanxiong (Chuanxiong), Hirudo (Shuizhi), and Pheretima (Dilong), among others. Modern pharmacological studies have confirmed that its core active ingredients (e.g., astragaloside IV, tanshinone IIA, ligustrazine) can ameliorate metabolic disorders through multiple mechanisms (22–26): On one hand, astragaloside IV can activate the AMPK pathway—a key energy sensor that not only enhances insulin sensitivity and inhibits hepatic gluconeogenesis but also exerts anti-inflammatory effects by suppressing NF-*κ*B signaling—thereby ameliorating metabolic disorders; tanshinone IIA can downregulate the expression of inflammatory cytokines (TNF-α, IL-6) in adipose tissue, alleviating IR. On the other hand, ligustrazine and hirudin can inhibit platelet overactivation, reduce hypercoagulability, and simultaneously improve vascular endothelial function and delay the formation of atherosclerotic plaques by modulating the endothelin-1 (ET-1)/nitric oxide (NO) balance. Preliminary studies have indicated that TJC has beneficial effects on both macro- and micro-vascular complications of diabetes, with a good safety profile (27–32); however, its impact on hard cardiovascular and cerebrovascular endpoints (e.g., myocardial infarction, stroke, cardiovascular death) remains unclear.

Currently, clinical research on interventions for cardiovascular and cerebrovascular risk in MetS predominantly focuses on Western drugs (e.g., GLP-1 receptor agonists, SGLT2 inhibitors) or single TCM components (e.g., Salvia miltiorrhiza injection). Large-scale, multi-center, long-term follow-up randomized controlled trials (RCTs) investigating TCM compounds, particularly Tongmai Jiangtang Capsule, are still lacking. The complexity and heterogeneity of MetS components, coupled with its insidious progression, necessitate multi-center collaboration to enroll populations from different regions and with diverse lifestyles, thereby enhancing the generalizability of the findings. Furthermore, the occurrence of cardiovascular and cerebrovascular events requires a long observation period. A double-blind design can mitigate observation bias and ensure the objectivity of efficacy assessment. Additionally, existing research on TCM compounds often focuses on short-term improvements in metabolic parameters, lacking comprehensive validation of the “metabolism-vascular-event” cascade, making it difficult to answer the core clinical question: “Can it ultimately reduce the risk of cardiovascular and cerebrovascular events?”

Therefore, conducting this study holds significant scientific and clinical value: Firstly, through a rigorously designed large-sample RCT, it aims to clarify the effect of TJC on cardiovascular and cerebrovascular events (including non-fatal myocardial infarction, non-fatal stroke, and cardiovascular death) in patients with MetS, filling the gap in high-level evidence for TCM compounds in this field. Secondly, it will systematically explore the potential mechanisms by which TJC improves cardiovascular and cerebrovascular risk, providing experimental support for its theory of “holistic regulation and multi-target intervention.” Thirdly, it will provide a new evidence-based approach for the integrated traditional Chinese and Western medicine treatment of MetS, promote the standardized application of TCM in chronic disease prevention and control, and contribute to achieving the comprehensive chronic disease prevention and control goals of “Healthy China 2030”.

In summary, this study addresses the global epidemic trend of MetS and unmet clinical needs, with the core objective of “verifying the impact of a glucose- and lipid-regulating TCM compound on hard cardiovascular and cerebrovascular endpoints.” Through rigorous design and scientific implementation, it is expected to provide new strategies for the prevention and treatment of MetS, offering significant social benefits and academic value.

## Methods/design

### Study objectives

The purpose of this study is to evaluate the effect of TJC on cardiovascular and cerebrovascular events in MetS patients with high cardiovascular risk. We hypothesize that TJC, as an add-on to standard Western medical treatment, can improve glycolipid metabolism and reduce cardiovascular and cerebrovascular events in patients with high cardiovascular and cerebrovascular risk and MetS (HCR-MetS). If the hypothesis holds, this study will provide clinical evidence for TJC in treating HCR-MetS.

### Study design

**Figure 1:**
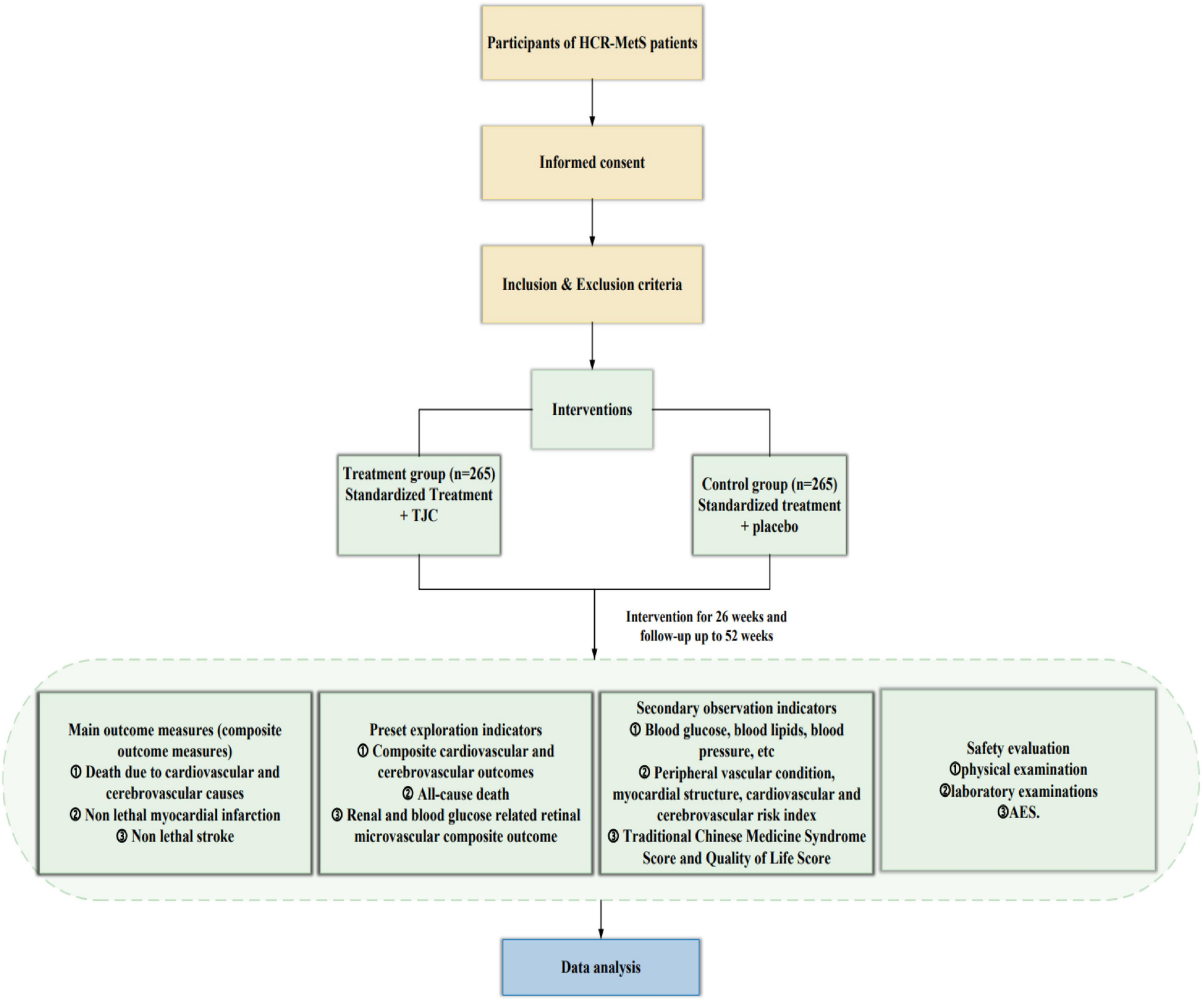
Schedule of the trial. HCR-MetS, high cardiovascular and cerebrovascular risk metabolic syndrome; TJC, Tongmai Jiangtang capsules; AES, adverse events.

This is a multicenter, randomized, double-blind, placebo-controlled, parallel-group superiority trial. The study protocol adheres to the Standard Protocol Items: Recommendations for Interventional Trials (SPIRIT) 2013 guidelines (18) and the CONSORT Extension for Chinese Herbal Medicine Interventions (19). The study was prospectively registered on ClinicalTrials.gov in April 2025 (NCT Number: NCT06908473). The study flow chart is shown in Figure 1.

### Setting and participants

The study will be conducted at six research sites in China (Guangdong Provincial Hospital of Traditional Chinese Medicine, Traditional Chinese Medicine hospital of Sichuan Province, Xiyuan Hospital of China Academy of Chinese Medical Sciences, Shanxi Provincial Hospital of Chinese Medicine, First Teaching Hospital of Tianjin University of Traditional Chinese Medicine, The First Affiliated Hospital of Guangzhou University of Chinese Medicine), located in southwest, northwest, central, north, and south China. Recruitment strategies will include advertisements on social media, as well as posters displayed at the six participating institutions. Recruitment will commence in May 2025 and is expected to be completed within 3 years. Patients who agree to participate will be examined and diagnosed by an associate chief physician to confirm their eligibility for the study.

### Eligibility criteria

#### Diagnostic criteria

MetS diagnostic criteria are based on the 2024 Chinese Guidelines for the Prevention and Treatment of Type 2 Diabetes Mellitus (16). Diagnosis requires the presence of three or more of the following criteria:

1. Abdominal obesity: waist circumference ≥90 cm for men, ≥85 cm for women.
2. Hyperglycemia: fasting plasma glucose ≥6.1 mmol/L or 2-hour postprandial plasma glucose ≥7.8 mmol/L and/or previously diagnosed diabetes under treatment.
3. Hypertension: blood pressure ≥130/85 mmHg and/or previously confirmed hypertension under treatment.
4. Fasting triglycerides (TG) ≥ 1.70 mmol/L.
5. Fasting high-density lipoprotein cholesterol (HDL-C) < 1.04 mmol/L.

#### Pattern diagnosis (syndrome differentiation) criteria for Qi deficiency and blood stasis

Diagnosis requires either (33):

At least one item from each of the main symptom categories ① and ②. Or at least two items from each of the secondary symptom categories ①, ②, and ③.

Main symptoms:

① Qi deficiency: shortness of breath, fatigue, lassitude, weak pulse.
② Blood stasis: stabbing pain, fixed pain, pain aggravated by pressure, tortuous vessels, subcutaneous ecchymosis, dark purple tongue or with ecchymosis/petechiae, engorged sublingual veins, choppy pulse, absent pulse, or deep-stringy, stringy-slow pulse.

Secondary symptoms:

① Qi deficiency: spontaneous sweating, reluctance to speak, pale tongue.
② Blood stasis: squamous and dry skin, limb numbness or hemiplegia.
③ Blood stasis: poor memory.

To ensure diagnostic consistency across the six multicenter sites and to minimize selection bias, the following procedure has been established. At each study site, three physicians with the academic rank of associate chief physician or higher will be responsible for TCM pattern diagnosis. Prior to the initiation of patient enrollment, these three physicians will independently evaluate 10 standardized case videos. Their diagnostic agreements will be assessed using Cohen's Kappa coefficient.

According to established benchmarks (34), a Kappa value of ≥0.75 is required to confirm “substantial to excellent” inter-rater reliability. Only after achieving this threshold will the center be permitted to start recruitment. If the initial Kappa value is <0.75, the physicians will undergo refresher training and repeat the assessment until the required consistency is met.

### Inclusion criteria

1. Age ≥65 years.
2. Meets the diagnostic criteria for MetS.
3. Meets the pattern diagnosis criteria for Qi deficiency and blood stasis.
4. High cardiovascular and cerebrovascular risk population, meeting any one of the following criteria (35):
  4.1. History of myocardial infarction.
  4.2. History of stroke or transient ischemic attack.
  4.3. History of coronary, carotid, or peripheral arterial revascularization.
  4.4. Coronary, carotid, or lower extremity arterial stenosis >50%.
  4.5. Positive exercise stress test or documented symptomatic coronary artery disease by any cardiac imaging, or unstable angina with ECG changes.
  4.6. Chronic heart failure (NYHA class II-III).
  4.7. Chronic kidney disease (eGFR <60 mL/min/1.73m^2^).

### Exclusion criteria

1. Secondary dyslipidemia, hypertension, or hyperglycemia.
2. Type 1 diabetes mellitus.
3. History of allergy to the study drugs.
4. Acute coronary or cerebrovascular event within the past 14 days.
5. Planned coronary, carotid, or peripheral arterial revascularization.
6. Chronic heart failure (NYHA class IV).
7. Undergoing continuous renal replacement therapy.
8. End-stage liver disease.
9. History of solid organ transplantation or on waiting list for solid organ transplantation.
10. Malignancy.

### Withdrawal criteria

1. Does not meet the inclusion criteria (found after enrollment).
2. Protocol violation regarding concomitant medication or failure to take study medication as prescribed, affecting outcome measures.
3. Incomplete data affecting the judgment of efficacy and safety.
4. Withdrawal from the trial for reasons other than adverse reactions or lack of efficacy.
5. Loss to follow-up, etc.

### Intervention

This is an add-on study protocol. All participants will receive standard guideline-directed medical therapy. The interventions are as follows:

Treatment group: TJC, 0.3 g/capsule, orally, three capsules per dose, three times per day. Control group: TJC simulant (placebo), 0.3 g/capsule, orally, three capsules per dose, three times per day.

The placebo capsules, composed of starch, bitter acid, and dextrin, among other excipients, are identical to the investigational drug in appearance, size, color, dosage form, weight, taste, and odor. They are manufactured by Baoding Buchang Tianhao Pharmaceutical Co., Ltd.

The drug composition of TJC is: Pseudostellariae Radix (Taizishen), Salviae Miltiorrhizae Radix Et Rhizoma (Danshen), Coptidis Rhizoma (Huanglian), Astragali Radix (Huangqi), Gynostemmatis Herba (Jiaogulan), Dioscoreae Rhizoma (Shanyao), Atractylodis Rhizoma (Cangzhu), Scrophulariae Radix (Xuanshen), Hirudo (Shuizhi), Malvae Verticillatae Fructus (Dongkuiguo), Puerariae Lobatae Radix (Gegen), etc. It is manufactured by Baoding Buchang Tianhao Pharmaceutical Co., Ltd.

Regarding standard treatment for MetS components and cardiovascular/cerebrovascular diseases during the intervention period, standard guidelines related to aspirin, statins, ACEIs or ARBs, metformin, SGLT2 inhibitors, and GLP-1 receptor agonists will be followed. Substitution with TCMs having similar components and/or effects to TJC is not permitted. Investigators must accurately record concomitant medications, and doses should be kept stable throughout the trial whenever possible.

### Study procedure

The schedule of study procedures is outlined in Table 1. The study will include a screening/baseline period, a treatment period, and a follow-up period. The screening/baseline period will last 2 weeks, the treatment period will be 26 weeks, and the follow-up period will be 26 weeks. Patients will be required to provide written informed consent after a complete explanation of the study during the screening period.

**Table 1:** Study schedule.

Research phase	Screening/baseline	Treatment phase			Observation phase after intervention
Number of visits	Visit 1	Visit 2	Visit 3	Visit 4	Visit 5
Visit time point	D-7–0	Week 4 ± 4 days	Week 12 ± 4 days	Week 26 ± 4 days	Week 52 ± 4 days
**Data collection**					
Written informed consent	□				
Inclusion/exclusion criteria	□				
Demographics	□				
Obtain central random number	□				
Previous history, medical history, and allergies	□				
Comorbidities and co-medications	□	□	□	□	□
Safety evaluation					
Vital signs and physical examination	□	□	□	□	□
Blood routine, Urine routine, Fecal routine	□		□	□	
Liver function(ALT, AST, ALB), kidney function(UN, Cr, UA, eGFR)	□		□	□	
12-Lead ECG	□		□	□	□
Efficiency evaluation					
Composite outcome measures	□	□	□	□	□
Preset exploration indicators	□	□	□	□	□
BMI, waist to hip ratio, body fat analysis	□	□	□	□	□
Blood glucose, blood lipids, blood pressure	□		□	□	□
Fasting insulin, C-peptide, HOMA-IR	□		□	□	□
Cardiovascular and cerebrovascular risk index	□		□	□	□
Troponin, cardiac enzymes, BNP	□		□	□	□
Vitamin D	□			□	□
UACR	□		□	□	□
Cardiac color doppler ultrasound	□			□	□
Neck vascular ultrasound	□			□	□
Traditional chinese medicine syndrome points	□	□	□	□	□
Quality of life rating	□	□	□	□	□
Omics analysis	□			□	
**Other jobs**					
Distribute experimental drugs	□	□	□		
Recall test drug		□	□	□	
Record AEs and combined medications		□	□	□	□
Compliance judgment		□	□	□	□

### Outcome measures

#### Primary outcome

Composite Endpoint (35)

1. Death due to cardiovascular and cerebrovascular reasons:
  1.1. Death due to cardiac arrest (with symptomatic myocardial ischemia, new ST-segment elevation or left bundle branch block, and angiographic or autopsy evidence of new thrombosis).
  1.2. Death due to cardiovascular causes: caused by acute myocardial Infarction (MI), heart failure, stroke, other cardiovascular causes, or without clear non-vascular cause.
  1.3. Sudden cardiac death: unexpected death due to cardiac causes in a previously stable condition.
  1.4. Death occurring within up to 30 days after a documented acute MI, without conclusive evidence of another cause; or death occurring based on clinical presentation and ECG evidence before biomarker confirmation of myocardial necrosis.
  1.5. Death occurring within up to 30 days after an intracranial hemorrhagic or non-hemorrhagic stroke, based on clinical presentation, neuroimaging, or autopsy, without conclusive evidence of another cause of death.
  1.6. Other cardiovascular or cerebrovascular causes of death.
2. Non-fatal myocardial infarction (MI):
  2.1. Spontaneous MI.
  2.2. ST-segment elevation MI (STEMI).
  2.3. Non-ST-segment elevation MI (NSTEMI).
  2.4. Percutaneous coronary intervention (PCI)-related MI.
  2.5. Coronary artery bypass grafting (CABG)-related MI.
  2.6. Silent acute MI.
3. Non-fatal stroke:
  3.1. Acute neurological dysfunction attributed to a vascular cause, documented by CT or MRI, with other causes excluded.
  3.2. Transient ischemic attack with symptoms lasting <24 h but with corresponding new imaging evidence (optional predefined criterion, might be moved to secondary depending on standard definitions).
  3.3. Micro-bleeds: small, round, hypointense lesions <5-10 mm on MRI (often considered a biomarker rather than a clinical event; might be more suitable for exploratory/system bio indicators).

### Pre-specified exploratory outcomes

1. Composite cardiovascular outcome (cardiovascular death, non-fatal MI, non-fatal stroke, coronary revascularization, or hospitalization for unstable angina or heart failure).
2. All-cause mortality.
3. Renal and glucose-related retinal microvascular composite outcomes.
  3.1. Renal composite outcome: New-onset persistent macroalbuminuria (UACR >300 mg/g), or persistent doubling of serum creatinine from upper limit of normal or eGFR <45 mL/min/1.73m^2^, or requiring continuous renal replacement therapy or death due to renal disease.
  3.2. Diabetic retinopathy microvascular composite outcome: Requiring retinal photocoagulation or intravitreal injections, vitreous hemorrhage, or blindness.

### Secondary outcomes

1. Metabolic and anthropometric parameters: blood glucose, lipids, blood pressure, BMI, waist-to-hip ratio, HOMA-IR, body composition analysis, Vitamin D.
2. Vascular and cardiac assessments: peripheral vascular status, myocardial status (left ventricular thickness, interventricular septum thickness), cardiovascular risk assessment indices (China-PAR score, Framingham Risk Score, QRISK3 score).
3. TCM symptom score and quality of life scores.

### Safety assessment

1. Safety evaluation indicators

Safety evaluation indicators include physical examination, vital signs (heart rate, respiration, temperature, blood pressure), laboratory tests, and adverse events (AEs), including severe adverse events (SAEs). Laboratory tests include complete blood count, urinalysis, blood biochemistry, and 12-lead ECG. Blood biochemistry will specifically include alanine aminotransferase (ALT), aspartate aminotransferase (AST), albumin (ALB), creatinine (Cr), blood urea nitrogen (BUN), and estimated glomerular filtration rate (eGFR).

During the treatment period, investigators must carefully observe and record all AEs and unexpected toxic side effects (including symptoms, signs, and laboratory abnormalities). Regardless of whether an AE is considered related to the investigational product, it must be documented in detail in the case report form (CRF), including time of onset, symptoms, signs, severity, duration, laboratory values, treatment administered and outcome, time elapsed, and follow-up time.

2. Clinical Events Committee and Independent Data Monitoring Committee

A Clinical Events Committee (CEC) comprising a cardiologist, a neurologist, and an imaging specialist has been established. The CEC will adjudicate all suspected cardiovascular and cerebrovascular endpoints (including 3P-MACE) and all serious adverse events (SAEs) in a blinded manner using pre-defined diagnostic criteria.

An Independent Data Monitoring Committee (IDMC) composed of a cardiovascular specialist, a neurologist, and a biostatistician has also been established. The IDMC is independent of the sponsor and investigators.

3. Pre-specified SAEs related to TJC components

In consideration of the Hirudo (Shuizhi) component, the following events are pre-specified as SAEs requiring immediate reporting and adjudication:① bleeding requiring platelet transfusion or hospitalization; intracranial hemorrhage; gastrointestinal bleeding requiring transfusion; clinically significant mucosal bleeding requiring medical intervention; ② prolongation of APTT or PT > 1.5× upper limit of normal (ULN); platelet count < 50 × 10⁹/L; ③ ALT or AST elevation > 3× ULN; eGFR reduction > 30% from baseline or < 45 mL/min/1.73 m^2^; serum creatinine doubling from baseline.

4. Blinded interim safety review

A pre-specified blinded interim safety review will be conducted when 265 participants (50% of the planned sample) have completed 12 weeks of treatment. The IDMC will review the incidence of SAEs that are possibly, probably, or definitely related to the investigational product, without being unblinded to treatment assignment. If the pooled incidence of drug-related SAEs exceeds a pre-defined safety threshold (based on historical data of TJC and similar herbal products), the IDMC may request unblinding of group assignments to assess whether the signal is disproportionate. The IDMC may then recommend early trial termination, protocol modification, or continuation. All recommendations will be documented and submitted to the ethics committee.

5. Emergency unblinding procedure

If a serious adverse event (particularly bleeding) requires knowledge of treatment allocation for clinical management, the investigator may request emergency unblinding through the trial coordinating center. Upon verification, the investigator will be informed of the patient's group assignment within 4 h. The reason, date, and outcome will be recorded in the CRF and reported to the IRB and IDMC within 24 h. All SAEs will be followed until resolution or stabilization.

### System biological indicators

This omics substudy is exploratory, aiming to identify potential mechanisms of TJC. Blood, urine, and stool samples will be collected from 30 participants per group from Guangdong Provincial Hospital of Traditional Chinese Medicine at baseline (Week 0) and post-treatment (Week 26), including fasting (10 h) blood samples and first-morning mid-stream urine and stool samples. The sample size (*N* = 30/group) is justified for exploratory omics studies (36, 37).

1. Biospecimen collection and processing

Blood will be collected into EDTA and serum separation tubes, centrifuged at 3,000 × g for 10 min at 4 °C within 2 h of collection to separate plasma, serum, and peripheral blood mononuclear cells (PBMCs). Urine will be centrifuged at 3,000 × g for 10 min at 4 °C to remove cellular debris, and the supernatant will be aliquoted. Stool will be collected using sterile containers, homogenized, and aliquoted into cryovials. All processing and centrifugation parameters are documented on dedicated log sheets.

2. Cryopreservation and biobanking

All processed biospecimens will be stored in the Guangdong Provincial Hospital of TCM Biobank—a certified biospecimen repository that operates under standardized management systems, has passed CNAS ISO 20387 accreditation for biospecimen quality and competency. The biobank is equipped with dedicated −80 °C freezers fitted with 24/7 temperature monitoring and alarm systems. A complete chain-of-custody log is maintained for each specimen, documenting all handling steps (collection, processing, storage, and transfer) with timestamps and responsible personnel.

3. Outsourced omics analysis

After sample collection is completed, all biospecimens will be transferred in a single batch to Shanghai Oebiotech Co., Ltd. for professional multi-omics analysis. Shipping will follow validated cold-chain protocols with real-time temperature recording and documented transfer logs. A pre-specified bioinformatics pipeline includes quality control, alignment, differential analysis, pathway enrichment, and multi-omics integration. No external validation is planned; internal QC (technical replicates, pooled QC samples, and coefficient of variation assessment) will ensure data quality.

### Sample size calculation

This is a two-arm, randomized, controlled superiority trial with a placebo control group and a TJC group. Using the composite primary outcome measure and a superiority test for proportions for sample size estimation. Based on literature, the estimated difference between the two groups is 15% (27, 38). With a one-sided *α*=0.025 and power (1-*β*) of 90%, using PASS 15 software for a 1:1 allocation ratio, and accounting for a potential 20% dropout rate (due to loss to follow-up, refusal, exclusion, etc.), a minimum of 265 subjects per group is required, resulting in a total sample size of at least 530 subjects.

### Randomization

Subjects who sign the informed consent form and meet the study criteria will be assigned a unique drug code via a central randomization system. Eligible subjects will be enrolled into groups sequentially according to time order and drug code. A computerized randomization schedule will be generated using the PROC PLAN procedure in SAS software. Study drugs will be coded according to the randomization schedule and then allocated to subjects. Note: Patients who fail screening but are suitable for re-screening must retain their original screening number. Drug codes for patients who are successfully screened and randomized but do not receive treatment cannot be reassigned to others. The next successfully screened patient will be assigned the next sequential drug code.

### Blindness

A double-blind design will be implemented. All investigators, subjects, physicians, drug administrators, and dispensing nurses will remain blinded to the treatment assignment until the study is completed and the database is locked. The blinding procedure will be managed by the clinical research unit leader, sponsor, and statistician. Unblinding will only be permitted in the event of a serious adverse event where knowledge of the treatment assignment is necessary for clinical management. The patient's group assignment will be obtained from the drug administrator.

Both TJC and its matching placebo are manufactured by Baoding Buchang Tianhao Pharmaceutical Co., Ltd. The main components of the TJC placebo are starch, bitter acid, and dextrin. It is identical to the active drug in appearance, size, color, dosage form, weight, taste, and odor. Furthermore, the quantity stated on the investigational product packaging will be blinded. The standard label for the investigational product will include the drug code, name of the clinical investigational product, indication, dosage and administration, treatment course, storage conditions, batch number, expiration date, and the supplier of the investigational product.

### Data collection and management

Investigators will enter raw data into the electronic CRF (eCRF) accurately and promptly based on the original observations from the subjects, strictly adhering to the trial protocol. To ensure data accuracy, two dedicated data entry personnel will perform independent double data entry and verification. Monitors will verify that all eCRFs are complete and consistent with the source data, querying any discrepancies as they arise. Researchers must promptly correct any errors or omissions identified.

### Monitoring

To further ensure clinical trial quality, the fundamental principles of “Quality by Design” and process control will be followed. Before the study begins, the sponsor will train all investigators and study coordinators at all centers to ensure consistency in drug administration and procedures. The sponsor will appoint monitors to conduct systematic monitoring of the trial in accordance with the principles of Good Clinical Practice (GCP), ensuring that the protocol is adhered to and that the data recorded in the CRFs are consistent with the source data.

### Statistical analysis

A detailed statistical analysis plan (SAP) will be finalized before database lock and unblinding. Statistical analyses will be performed using SAS version 9.4 (SAS Institute Inc., Cary, NC, USA) by the statistics department of the Clinical Research Center at Guangdong Provincial Hospital of Traditional Chinese Medicine. Data from all randomized patients will be analyzed according to the Intent-to-Treat (ITT) principle. Consistent with the CONSORT statement and ITT principle, the last observation carried forward (LOCF) method will be used to handle missing data for the primary analysis.

① Hypothesis Testing: All statistical tests will be two-sided, with a *p*-value less than 0.05 considered statistically significant.② Statistical Description: Normally distributed continuous variables will be described using “mean ± standard deviation,” non-normally distributed continuous variables using “median (interquartile range),” and categorical data using frequencies and percentages.③ Choice of Statistical Methods: Appropriate statistical methods will be selected based on the nature of the data (continuous, categorical, ordinal). Categorical data will be compared using the Chi-square test or Fisher's exact test. For continuous data meeting normality and homogeneity of variance assumptions, the t-test will be used for between-group comparisons; otherwise, the Wilcoxon rank-sum test will be employed. Ordinal data between two groups will be compared using the Mann–Whitney U test, and for multiple groups, the Kruskal–Wallis test. The center effect in the multi-center efficacy evaluation will be analyzed using the Cochran-Mantel-Haenszel method.④ Statistical Analysis Steps: The statistician will follow the pre-specified SAP, meticulously check and confirm the clinical trial data, and then proceed with the statistical analysis. This will include descriptive statistics, baseline comparison of clinical characteristics before treatment, efficacy analysis, safety evaluation, and analysis of excluded and dropped-out cases. If potential confounding factors that are difficult to fully control or were not controlled (e.g., age, disease severity, duration) are imbalanced between groups at baseline, analysis of covariance (ANCOVA) will be used to analyze the impact of these factors on the between-group differences in efficacy, thereby adjusting for the influence of confounders.

### Current status

Patient recruitment for the trial is scheduled to commence in May 2025. The study protocol was successfully registered on ClinicalTrials.gov (https://register.clinicaltrials.gov) on April 2, 2025. The trial registration number is NCT06908473.

## Discussion

Driven by the high prevalence of MetS, its associated elevated cardiovascular and cerebrovascular risk, and the current lack of clarity regarding the effect of Tongmai Jiangtang Capsule—a glucose- and lipid-lowering agent—on cardiovascular events in MetS patients, there is an urgent need to fill the gap in high-level evidence for TCM compounds in this domain, prompting the initiation of this study. The adoption of a multi-center, double-blind RCT design represents the current “gold standard” for validating intervention efficacy. The multi-center design, by enrolling patients from diverse geographical regions (North, East, South, Southwest, Northwest China) and lifestyles (urban/rural), significantly enhances sample representativeness and reduces the “selection bias” inherent in single-center studies. Compared to other TCM studies, the multi-center nature of this trial better aligns with the characteristics of MetS, which involves “multi-system involvement and high population heterogeneity,” thereby strengthening the generalizability of the findings.

Notably, this study sets the primary endpoint as 3P-MACE (including non-fatal myocardial infarction, non-fatal stroke, and cardiovascular death). This “hard endpoint” directly reflects clinical outcomes and holds greater clinical significance compared to surrogate markers commonly used in traditional studies (39) [e.g., carotid intima-media thickness [IMT], flow-mediated dilation [FMD]]. The secondary endpoints encompass metabolic parameters and vascular-related indicators, addressing the developmental cascade of MetS: “metabolism-vascular injury-cardiovascular events.” This “stratified endpoint design” breaks through the limitation of traditional TCM research that often “emphasizes symptoms over hard outcomes,” better aligning with the core requirement of modern evidence-based medicine for demonstrating “clinical value.”

TJC has been marketed for years. Previous clinical trials and observational studies indicate that its adverse reactions are primarily mild gastrointestinal discomfort, with no reports of severe hepatic or renal impairment, ensuring good patient tolerability. Multiple clinical studies suggest that TJC can improve insulin resistance, effectively alleviate diabetic neuropathy and nephropathy, and improve prognosis in diabetic patients with cerebral infarction (32, 40).

The ten key pharmacologically active components in TJC, in order, are: quercetin, eugenol, campesterol, β-sitosterol, isofucosterol, stigmasterol, kaempferol, luteolin, isorhamnetin, and hederagenin. Among these, quercetin can effectively prevent and improve diabetic complications through multiple signaling pathways, including TNF-α, NF-κB, AMPK, AKT, and NRF2 (41–43). These pathways collectively contribute to anti-inflammatory and vascular protective activities (44, 45). β-sitosterol can enhance pancreatic β-cell function, increase insulin secretion and activity, effectively promote glucose uptake in adipocytes, and regulate fat synthesis and decomposition (46–48). Stigmasterol can promote glucose uptake in L6 cells *in vitro* and reduce fasting blood glucose and lipid levels in KKAy mice *in vivo*, alleviating insulin resistance and improving oral glucose tolerance (49–51). Kaempferol can upregulate the expression of genes and proteins related to the PI3K-AKT-GLUT4 signaling pathway in skeletal muscle, reduce blood glucose and body weight in KKAy mice, improve insulin resistance, and possesses significant anti-inflammatory activity (52–54). Eugenol's mitigation of cerebrovascular spasm may be related to inhibiting apoptosis and inflammatory responses within the basilar artery (55, 56). Stigmasterol, campesterol, and isofucosterol are all plant sterols, which can significantly reduce total cholesterol and LDL-C levels, increase HDL-C levels, and improve thickening of the aortic intima and media as well as smooth muscle cell migration (57–59). Meanwhile, quercetin, kaempferol, luteolin, and isorhamnetin are flavonols, possessing anti-inflammatory, antioxidant, cardiovascular, and neuroprotective effects (60–62). This suggests that beyond its established effects on improving insulin resistance and lowering blood glucose, TJC also has lipid-regulating, anti-inflammatory, and vascular compliance-improving properties, implying potential benefits for reducing cardiovascular risk. However, the actual clinical effect requires confirmation through high-quality RCTs.

If this study confirms that TJC significantly reduces the risk of cardiovascular and cerebrovascular events in MetS patients, its clinical significance will be manifold: Firstly, it will provide a new strategy for the “integrated traditional Chinese and Western medicine treatment” of MetS—addressing the shortcomings of current regimens in improving endothelial function and inhibiting chronic inflammation. Secondly, it will advance the “modern evaluation of TCM compounds"—using “high-level evidence” from RCTs to challenge the stereotype of “ambiguous efficacy of TCM” and provide evidence-based support for the clinical application of TCM compounds. Thirdly, it will contribute to the “Healthy China 2030” goals—by reducing the rate of cardiovascular events in MetS patients, decreasing healthcare resource consumption, and improving patients’ quality of life.

Even if the study results do not meet the primary endpoint (e.g., no significant difference in event rates between groups), the in-depth analysis of TJC's mechanisms of action (e.g., showing benefit only in subgroups with severe IR or high inflammatory burden) will provide important clues for subsequent formula optimization (e.g., increasing the dose of Astragalus to enhance AMPK activation) and conducting “precision intervention” studies (e.g., subgroup selection based on biomarkers such as HOMA-IR).

In conclusion, through its rigorous design, multi-dimensional endpoint setting, and scientific strategies for addressing challenges, this study is expected to provide high-level evidence for the prevention and treatment of cardiovascular and cerebrovascular events in MetS. Simultaneously, it offers a replicable “demonstration template” for the modern research and clinical application of TCM compounds. Its results are significant not only for the clinical value of TJC but also for providing crucial support for the national strategy of “Integrated Traditional Chinese and Western Medicine for Chronic Disease Prevention and Treatment.”

## Conclusion

This study will investigate the therapeutic potential of TJC as a combination drug. The results of the study will provide reliable clinical research evidence for the application of TJC in improving cardiac function and exercise tolerance in MetS patients.
